# A Review on Integrated ZnO-Based SERS Biosensors and Their Potential in Detecting Biomarkers of Neurodegenerative Diseases

**DOI:** 10.3390/bios13050499

**Published:** 2023-04-25

**Authors:** Alia Colniță, Vlad-Alexandru Toma, Ioana Andreea Brezeștean, Muhammad Ali Tahir, Nicoleta Elena Dina

**Affiliations:** 1 Department of Molecular and Biomolecular Physics, National Institute for Research and Development of Isotopic and Molecular Technologies, 67-103 Donat, 400293 Cluj-Napoca, Romania; 2 Department of Molecular Biology and Biotechnology, Faculty of Biology and Geology, Babeș-Bolyai University, 5-7 Clinicilor, 400006 Cluj-Napoca, Romania; 3 Institute of Biological Research, Department of Biochemistry and Experimental Biology, 48 Republicii, Branch of NIRDBS Bucharest, 400015 Cluj-Napoca, Romania; 4 Shanghai Key Laboratory of Atmospheric Particle Pollution and Prevention, Department of Environmental Science & Engineering, Fudan University, Shanghai 200433, China

**Keywords:** neurodegenerative diseases, SERS, zinc-oxide-based SERS substrates, biomarkers, dopamine, amyloid-β, diagnosis

## Abstract

Surface-enhanced Raman spectroscopy (SERS) applications in clinical diagnosis and spectral pathology are increasing due to the potential of the technique to bio-barcode incipient and differential diseases via real-time monitoring of biomarkers in fluids and in real-time via biomolecular fingerprinting. Additionally, the rapid advancements in micro/nanotechnology have a visible influence in all aspects of science and life. The miniaturization and enhanced properties of materials at the micro/nanoscale transcended the confines of the laboratory and are revolutionizing domains such as electronics, optics, medicine, and environmental science. The societal and technological impact of SERS biosensing by using semiconductor-based nanostructured smart substrates will be huge once minor technical pitfalls are solved. Herein, challenges in clinical routine testing are addressed in order to understand the context of how SERS can perform in real, in vivo sampling and bioassays for early neurodegenerative disease (ND) diagnosis. The main interest in translating SERS into clinical practice is reinforced by the practical advantages: portability of the designed setups, versatility in using nanomaterials of various matter and costs, readiness, and reliability. As we will present in this review, in the frame of technology readiness levels (TRL), the current maturity reached by semiconductor-based SERS biosensors, in particular that of zinc oxide (ZnO)-based hybrid SERS substrates, is situated at the development level TRL 6 (out of 9 levels). Three-dimensional, multilayered SERS substrates that provide additional plasmonic hot spots in the z-axis are of key importance in designing highly performant SERS biosensors for the detection of ND biomarkers.

## 1. Introduction

The neurodegeneration spectrum takes into account the contribution of several molecular sciences in disease definition and features. However, several canonic neurodegenerations such as Parkinson’s disease (PD) or Alzheimer’s disease (AD) are references in neuroscience as NDs. According to the “neurogenesis hypothesis of depression”, major depressive disorder (MDD) can also be considered a mild neurodegenerative disorder that is caused by changes in the rate of neurogenesis [1,2,3]. Other classes of disorders known as neurodevelopmental diseases such as autism spectrum disorders (ASDs) may induce neurodegeneration in the form of neuronal cell loss, activated microglia and astrocytes, proinflammatory cytokines, oxidative stress, and elevated 8-oxo-guanosine levels [3].

From the medical point of view, it is still not completely understood what neural mechanisms should be monitored; however, the main pathways are sketched by experts. Traditional testing for NDs mainly relies on psychiatric queries, biomedical imaging methods such as computer tomography (CT) or hyperspectral imaging, and usually offers a tardive diagnosis. Invasive tests based on the thorough analysis of cerebrospinal fluid (CSF), such as the common lumbar puncture, are often disliked by patients [4]. Thus, current clinical trials are designed based on the stringent need for non-invasive and early diagnosis of NDs coupled with the drive for efficient mechanisms to probe biomarkers predicting disease onset (prognosis).

To keep track of the recent advancements and breakthroughs in the societal environment, the need for new diagnosis methods for NDs is of extreme importance. Since the 2018 addition of the Alzheimer’s Association’s International Conference (Chicago, IL, USA) [5], new diagnosis recommendations were introduced for rapid screening and prognosis tools, accurate clinical management, and a correct assessing of the genetic implications by participating in clinical trials. The impact and costs that NDs create for public health systems are huge [6]; therefore, there is a crucial need for fast and reliable alternative diagnostic tools.

Many NDs are considered proteinopathies, and there is much interest in tackling their associated pathomechanisms along with the pathophysiological role of relevant biological markers [7]. The most targeted bioanalytes for ND diagnosis are: (i) neurotransmitter-related metabolites present in biofluids (such as CSF), with specific levels in case of neural deterioration, or (ii) biomarkers revealed by the histopathology of brain tissue. Herein, we discuss the potential of Raman/surface-enhanced Raman scattering (SERS) spectroscopic techniques to offer reliable solutions in current clinical practice. The recent improvements in substrate design and rational fabrication expanded the use of SERS-based biosensors in biological and biomedical applications, such as detection of nuclear acids, biomarker monitoring, or medical diagnosis [8,9,10].

Neurotransmitter-related CSF metabolites have been mainly targeted in SERS-based biosensing as they can be detected prior to the cognitive symptoms’ onset. These metabolites are considered useful predictive tools and good biomarker candidates for ND detection having been recently identified and summarized by Wakamatsu et al. [11]: amyloid-β (*Aβ*) peptides [12,13], total (T) or phosphorylated (P-tau) tau proteins, α-synuclein (αSyn), neurogranin, neuroligin-1, miRNAs, and γ-aminobutyric acid (GABA). Melatonin, serotonin, glutamate, dopamine, norepinephrine, and epinephrine are also of interest [14] and have been detected using SERS biosensors down to picomolar (pM) [15,16,17,18,19] or even attomolar concentrations when using solid-state SERS platforms [20,21].

Before acknowledging in vivo monitoring approaches with imaging performance (magnetic resonance imaging, optical coherence tomography, two-photon excited fluorescence, etc.), we will discuss the currently developed rapid and sensitive SERS biosensors for the early diagnosis of NDs. Major unmet needs still exist, mainly related to the real-time detection and quantification of peripheral ND-related biomarkers (genomics and proteomics) in CSF, blood, or urine at concentrations as low as the femtomolar (fM) level. In this review, based on the scarce existing studies, we assess the possibility for integrated ZnO-based SERS biosensors to be considered as emerging valuable tools in ND diagnoses with real chances to be implemented as routine prognosis/diagnosis methods.

## 2. Conventional Approaches for ND Diagnosis

Dementia is considered to be an “umbrella term” to describe a neurodegenerative, progressive condition characterized by “functional” impairment. It is mostly irreversible due to the degeneration of brain cells and their interconnections, which compromises the daily activities of a person’s life. AD is the most prevalent form of dementia and is thought to account for 70–80% of cases in the elderly [22]. Initially, AD manifests with an impairment of recent memory function and attention and continues with failure of language skills, visual–spatial orientation, abstract thinking, and judgment. The diagnosis of AD currently relies on the identification of characteristic clinical signs and can only be confirmed by the distinctive cellular pathology evidence from postmortem examination of the brain [23].

The histological changes described so far involve three aspects: (i) collections of neurofibrillary tangles, (ii) pericellular deposits of amyloid, and (iii) a diffuse loss of neurons. These changes are most frequent in the neocortex, hippocampal formation, amygdala neurons, and basal forebrain nuclei [24]. A mutation of the gene encoding amyloid precursor protein (APP) has emerged as a prominent candidate because of both the significant amyloid deposits in AD and the isolation of a fragment of APP, *Aβ*, from amyloid plaques [25,26,27,28]. In this light, using enzyme-linked immunosorbent assay (ELISA) or protein immunoblot (Western blot) are considered conventional molecular methods for testing blood or CSF samples for *Aβ* peptide in AD diagnosis [29,30]. In addition to *Aβ*, two other proteins were associated with the genetic induction of AD, presenilin 1 and presenilin 2 [31]. Mutations of presenilin 1 and 2 modify the processing of APP, resulting in increased amounts of *Aβ42*, a particularly toxic form of the *Aβ* peptide [31,32]. Accumulation of the *Aβ42* peptide, in particular, is thought to be a key factor. However, this can be established by immunohistochemistry and Western blot analyses, procedures that are performed in research experimental conditions or on postmortem brain samples. In terms of accuracy–LOD–costs, these methods provide average (immunohistochemistry) to high accuracy (Western blot) but are also expensive and research laboratory dependent.

The second most common neurodegenerative disease is considered to be PD, which predominantly affects the “dopaminergic” neurons located in the substantia nigra part of the brain [33]. It has a relatively long onset period during which symptoms such as tremor at rest, bradykinesia, rigidity of the extremities and neck, and minimal facial expressions [34] are experienced by the patient. Currently, joint genetic and environmental factors (pesticide, herbicide, and heavy metal exposure) are thought to cause the progressive deterioration of these dopaminergic neurons [35].

Besides MRI, the conventional diagnosis of PD relies solely on the clinical signs. Recent advances have suggested a few molecular approaches to detect PD using biomarkers in the blood or CSF [36,37,38]. These analyses involved gene mutations for α-synuclein, Parkin, and DJ-1 genes that were noted as important in rare forms of PD [39,40]. Another proposed method to diagnose PD was the estimation of the dopamine concentration in CSF with chromatography; however, this procedure is inconsistent in terms of results. The measurement of tyrosine hydroxylase activity in different fluids such as plasma, serum, or cerebrospinal fluid has also been considered for PD diagnosis [41,42]. Classical PD diagnosis based on imaging techniques is still the gold-standard procedure, whereas other methods are inconsistent or very expensive [42]. Other time-dependent evolutive diseases are considered such as autism spectrum disorders (ASDs) and major depressive disorders (MDDs).

Autism spectrum disorders (ASD) are developmental disorders that result from an abnormal process of brain development and maturation [43,44]. The underdevelopment of the brain manifests in impairments in social interaction, language, communication and imaginative play, and in the range of interests and activities. Along this line, many therapeutic approaches were adopted in order to ameliorate the autism-related symptoms and to improve the magnitude of social skills in ASD. One of the most popular approaches used in the treatment of autism is the Social Story^TM^ method [45]. This type of intervention aims at providing ASD individuals with the social information they lack and thus help them to develop appropriate behavior in their social life. Recently published studies suggest a connection between ASD risk genes and certain proteins involved in the synaptic mechanisms (GABA and glutamate receptors such as GRIN2B) or membrane ion channels and genes coding for proteins involved in cell regulation [46]. Furthermore, insights into the neuropathology in ASD have revealed abnormal cellular changes in the limbic system structures, an increased packing density of neurons in the pyramidal layers of the hippocampal and subicular subfields, and a reduction in the density and distribution of GABA receptors [47]. Consistent abnormalities on the long arm of chromosome 15 in the q11-13 region that codes for three isoforms of GABA receptor subunits have also been reported [48]. Moreover, early brain-derived neurotrophic factor (BDNF) hyperactivity may play an etiological role in autism as serum BDNF levels positively correlate with the cortical BDNF levels and have increased values in autism [49]. Currently, ASD diagnostic and clinical trials selection criteria are heavily guided by the Diagnostic and Statistical Manual of Mental Disorders or behavioral diagnostic scales [50]. Furthermore, the genetic underpinnings of ASD had a successful diagnostic rate of up to 38% by introducing molecular biology methods (DNA sequencing, transcriptomics, and polymerase chain reaction—PCR, RT-PCR) [51,52,53]. Moreover, the investigation of several molecular panels (e.g., RAREs, blood exosomes with brain trophic factors, miRNAs, or neurotransmitters) that are closely related to ASD is a current diagnostic procedure. Unfortunately, these molecular-based protocols come with a high financial impact.

Structural magnetic neuroimaging methods such as proton magnetic resonance spectroscopy (^1^H-MRS) can successfully quantify in vivo the low-molecular-weight molecular metabolites related to ASD in children, such as creatinine, phosphocreatine (Cr + PCr), N-acetylaspartate (NAA), choline, myo-inositol, and lactate at clinical and sub-clinical detection levels. The pros and cons of these are detailed in the review by Ford et al. [54]. On the other hand, functional MR imaging offers valuable information on aspects regarding social skills and independent domestic activities management [55]. It is also worth mentioning an attempt to develop a complementary spectroscopic diagnostic tool based on a chemometric model that relies on infrared (IR) spectroscopic data for ASD detection in children and teenagers 4–17 years old [56]. Principal component analysis (PCA)- and partial least squares discriminant analysis (PLS-DA)-based models analyzing FT-IR spectra recorded on blood serum samples have shown a clear separation of ASD individuals from healthy control samples.

On the other hand, MDD is characterized by long-lasting desensitization of 5-HT1A autoreceptors in the dorsal raphe [57]. Other changes have also been detected, such as changes in the neurotrophins NT3, NT4, and BDNF, as well as increased corticosterone and IL34 levels in the blood during MDD [58,59]. Brain epitranscriptomic studies revealed a series of downregulated miRNAs (miR-96, 414, 182, 193, 298, and 429) in MDD [60] that participate in the alterations of the gene expression network in the hippocampus, amygdala, and prefrontal cortex. In addition, recent studies [61,62,63] have emphasized miR323a’s important role in the development of MDD and the possibility to exploit it as a therapeutic target. Moreover, brain miRs involved in MDD are conveyed intercellularly through extracellular vesicles (EVs) generated in multivesicular bodies (MVB), and EVs pass the blood–brain barrier and circulate in the blood flow with their cellular-origin tags (L1CAM for neurons (unspecific), GFAP for astrocytes, and CNP for oligodendrocytes) [64,65,66]. Based on experimental studies with animal models, EV isolation from blood plasma in MDD subjects can be developed as an accurate predictive test based on analyzing miR323a as a specific biomarker.

Thus, improving molecular-based diagnosis techniques will definitely add value to the current advances in terms of diagnosis, prognosis, and therapeutic strategies and could pinpoint the biological signature of NDs in clinical settings. Table 1 summarizes the main techniques currently used for ND diagnosis by highlighting their advantages and limitations in clinical practice.

## 3. Raman and SERS-Spectroscopy-Based Monitoring of Main ND Biomarkers

Raman spectroscopy (RS) is an omnipresent, molecular-specific technique used for the identification of chemical species or their structural or functional properties. The specificity of the Raman scattering process is given by the frequency of the scattered light resulting from the inelastic interaction of the laser with the sample, which is associated with a unique molecular fingerprint. It is recommended as a promising alternative to conventional methods for early diagnosis due to inherent key assets: no labelling is required, less sample preparation involved and it is non-destructive for the sample, it has real-time and in vivo monitoring capabilities, it can be combined with imaging techniques, it is non-invasive, and it is considered to be a cost-effective approach. Furthermore, the minimal interference from water with the fingerprint region makes it very suitable for in situ biomolecular sample analysis and detection in various medical fields. For instance, it was used as an effective diagnosis tool for oral diseases such as dental caries, oral cancer lesions [69,70], gingivitis, or periodontitis [71]. The ability of RS to accurately and non-invasively identify the biochemical differences between the healthy and pathological statuses of tissues made it very popular in the diagnosis of different types of cancer [71,72,73,74,75,76].

In the case of the diagnosis and prognosis of NDs, RS in combination with multivariate tools has been proven an effective tool [68] to develop, for instance, a classification model for the diagnosis of AD based on the intensity levels of carotenoids from blood serum samples [77]. Despite the attributes that make RS a promising diagnostic tool, it does have several drawbacks that limit its translation into clinical use: the small cross section, weak Raman signal that imposes long acquisition times and sample degradation [75]. The Raman signal is dependent on the concentration of the analyte, making it a valuable analytical technique for molecular species’ quantification but with low sensitivity.

The currently reported strategies for Raman monitoring of ND biomarkers and prognosis/diagnosis for the onset of NDs are based on:

- Closely monitoring signal fluctuations related to the accumulation/aggregation of specific proteins: cellular prion protein (PrP^C^)—a cell surface glycol protein attached to the plasma membrane—levels from cells [78], *Aβ* peptides, αSyn, tau protein, etc.;
- Identifying functional molecular groups associated with NDs [79,80];
- Dopamine level monitoring from biofluids for early PD diagnosis [81,82,83] and for HD diagnosis at pre-installing stages;
- Raman fingerprinting for different classes of components in tissular samples (lipids, proteins, and β-sheets) and their simultaneous quantification [13,78,84,85,86].

**Figure 1:**
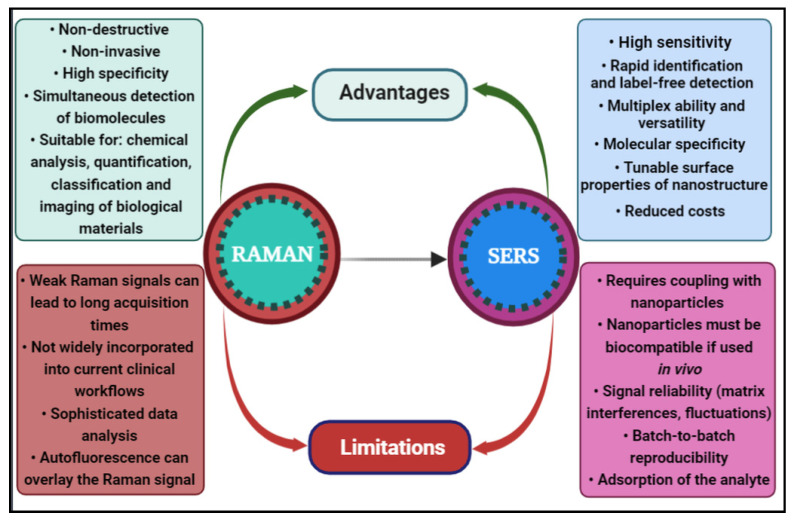
The advantages and limitations of the Raman-based vs. SERS-based approaches for ND diagnosis and monitoring.

In comparison with RS, surface-enhanced Raman scattering (SERS) has developed into a high-throughput detection technique with a wider range of applications, including clinical diagnosis [9,10,87,88], due to the fabrication and synthesis of a wide range of substrates [89,90]. A schematic view of the advantages and inherent downsides when selecting Raman or SERS as a diagnosis tool for NDs is shown in Figure 1.

**Figure 2:**
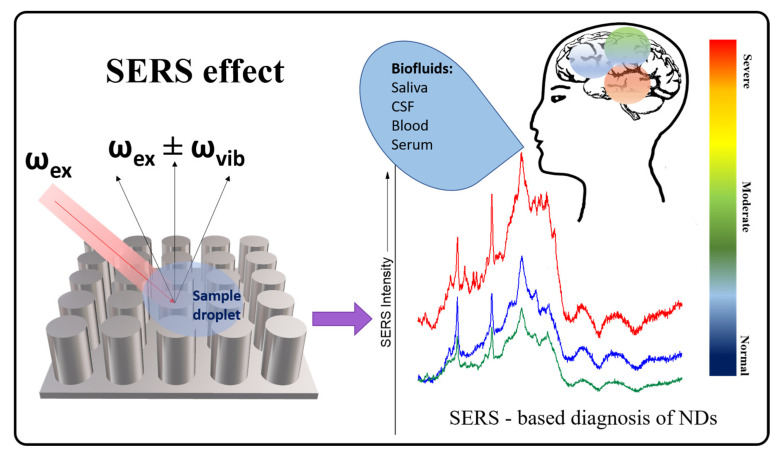
The principle of the SERS effect and the sequential steps for SERS-based diagnosis of NDs from spectra acquisition to data analysis and spectral pathology (prognosis, early diagnosis, and stage diagnosis).

SERS spectroscopy exploits the plasmonic oscillations that generate an enhanced electromagnetic field in the surroundings of metallic nanostructures interacting with (bio)molecules under irradiation with a laser [91]. The intensity of this field decreases dramatically with the distance from the nanostructured surface used as enhancer. Moreover, the enhancement mechanism is considered dual: combined with the electromagnetic effect (EM) there is a chemical contribution (CM) consisting of the formation of an analyte–metal assembly, with the condition of physi-/chemisorption of the molecules to the metallic substrate [92]. Hence, SERS spectroscopy becomes an important analytical tool with high quantitative output given by the electromagnetic enhancement and an excellent qualitative control rendered by the molecular specificity and chemical enhancement. Figure 2 shows the SERS effect principle, based on the scattered light detection, collected after the molecule–substrate interaction takes place [93].

Compared with conventional trace-level sensing by, for instance, high-performance liquid chromatography coupled with mass spectrometry (HPLC-MS), SERS is favorable in terms of the overall low cost per analysis and easier sample preparation, which renders the technique faster, simpler, and equally ultrasensitive.

An overview of the main ongoing strategies used to develop SERS-based biosensors is provided in [94], focusing on the: micro/nanofabrication of reproducible SERS-active substrates [95] or the design and synthesis of SERS nanotags and their integration in portable high-throughput platforms for point-of-care (PoC) testing [9,10]. SERS applications in clinical diagnosis and spectral pathology are increasing due to the techniques potential to bio-barcode incipient and differential diseases by real-time monitoring of biomarkers in fluids via biomolecular fingerprinting [90].

Herein, challenges in clinical routine testing are addressed in order to understand the context of how SERS can perform in real in vivo sampling and bioassays for early ND diagnosis. The main interest in translating SERS into clinical practice is related to the fact that high-performance SERS substrates enable detection of clinically relevant compounds with ultrasensitivity, up to 10 orders of magnitude greater than other Raman techniques [68,96]. Moreover, vibrational spectroscopic fingerprinting is sensitive to protein misfolding and aggregation, enabling specific identification of proteinopathies [7,68,97]. Spectral differences could reveal specific bands as indicators for healthy or diseased cells [98,99,100] (Figure 2).

In the following, a comprehensive survey of the last decade’s timeframe is offered considering the main high-throughput SERS biosensors used in practice for ND diagnosis.

## 4. SERS Biosensors Developed for Proteinopathies

SERS technology is exploiting a wide area of nanomaterials with proven potential towards applications in diagnosis [10,101,102] and monitoring processes for chronic diseases [99,103] as a part of the nanomedicine area. AD diagnosis is mostly based on the ultralow detection of the tau protein, which is associated with neurofibrillary tangles, or the *Aβ* peptide as main component of the extracellular plaques, the early indicator for disease onset. In this direction, recently designed strategies for early AD diagnosis focus on testing brain tissue [104,105], CSF [83], blood [106], or serum [107] samples for abnormal levels of certain biomarkers.

Practically, the main biomarkers were profiled by SERS via antibody-coated NPs for direct detection of *Aβ* [108] and tau [109]. Furthermore, there were SERS-based label-free approaches that were successfully tested for the dynamic processes of *Aβ* that lead to the aggregation and subsequent fibrillation in AD pathology [110]. Thus, *Aβ*’s dynamic monitoring and quantitative detection in biofluids is considered one of the most efficient methods for early diagnosis of AD. Once peptides are adsorbed onto the metallic surfaces, structural rearrangement processes take place that can be monitored by SERS; however, the effect of the nano-sized metallic structures is still controversial. Moreover, peptide size is crucial for adsorption to the surface and further SERS enhancement of its specific fingerprint. If the polypeptide chain length limits the short-range (few nanometers) effect of the strong SERS optical response, then the protein spectral profile is substandard. Creating an ideal contact for the sample with the metallic surface might require the use of super hydrophobic surfaces [111], dehydration, or drop-coating deposition followed by laser irradiation.

In terms of biosensing, great progress was made by the behavior detection biosensors that can predict or diagnose the onset of NDs, such as AD, by a non-invasively monitoring body motion, eye movement, speech, or even multifunctional indicators at home, at the desk, or in clinical setting [112]. Multi-sensor-based intelligent home monitoring or wearable devices able to track body functions relevant for ND prognosis seem to be the future trend in wellbeing and mental healthcare. More elaborated immunoassays are reported for electrochemiluminescence [113] or electrochemical [114,115] ultralow ex vivo *Aβ* or prion protein detection (from 0.1 ng/mL up to nanomolar levels) and for SERS detection below fM concentrations for the tau protein [109].

**Figure 3:**
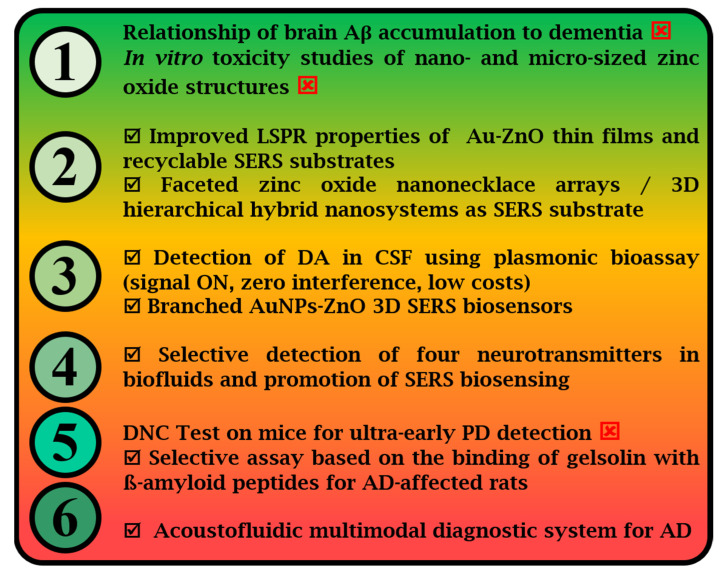
The main progress steps on the technology readiness levels (TRL) road to alternative approaches for ND diagnosis, SERS-based (☑) or not (⊠).

We resume our survey with attempts based on spectroscopic monitoring of neurotransmitters ex vivo (as isolated samples) by using SERS performant solid substrates consisting of Au [20,116,117], Ag [107,110], semiconductor [118,119,120], or hybrid [13,121] layered nanostructured surfaces. We start by outlining the relevant biomarkers for early detection when using highly performant SERS-based biosensing platforms and the subsequent practical challenges. Figure 3 shows the technological progress in terms of ultralow detection of those biomarkers and eventual evaluation of disease stage.

### Relevant Biomarkers Detected in SERS-Based Diagnosis of NDs

Prion protein. Copper-binding ability of this protein is a key aspect in its normal functioning (capturing copper ions from the extracellular premises) and can be used as a selective spectral feature from the abundance of Raman bands of a cell (amides, lipids, nucleic acids, and so on). Monitoring using Raman/SERS of the cell lines that express PrPC at different levels has already been demonstrated as a diagnostic tool for predisposition to prion disorders [98]. By using nano-sized Au-based nanostructures, the affinity of the prion protein to bind to copper ions in low concentrations was assessed in vitro, with low costs [98].

α-Synuclein (presynaptic protein). It has a strong propensity to form α-helical structures and has specific Raman spectral features at 1320 cm^−1^ and 1675 cm^−1^. It is difficult to sense by traditional means due to its versatile and dynamic conformation that is specific to the predominant polyproline II helix (PPII). By monitoring the amide I region, it is possible to distinguish between α-helices (1650–1656 cm^−1^), β sheets (1664–1670 cm^−1^), and unordered secondary structures (1675–1680 cm^−1^) using Raman analysis [122]. This would greatly help in PD diagnosis from saliva samples because it has been undetectable in urine so far.

**Figure 4:**
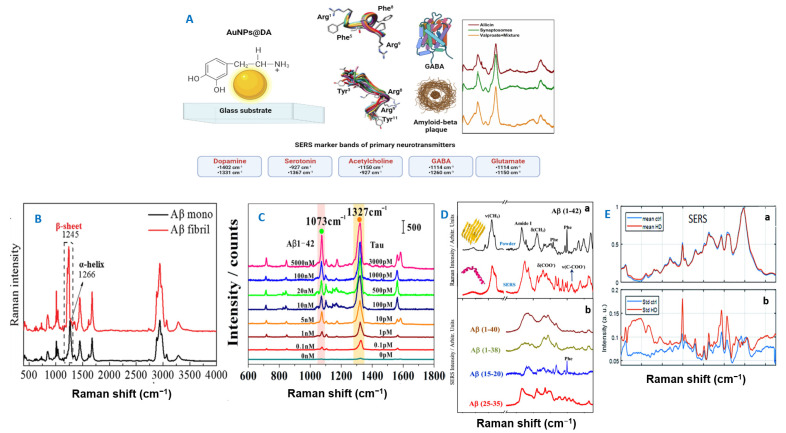
(**A**) The most targeted neurotransmitters for ND diagnosis in current practice are depicted. (**B**) Label-free SERS spectra of chiral triangular Au nanorings fabricated with a platinum (Pt) framework—d-Pt@Au TNRs with *Aβ*42 monomers and fibrils. Reproduced with permission [124]. Copyright 2021, Wiley-VCH GmbH. (**C**) Typical SERS signals obtained in response to different concentrations of Tau protein and *Aβ*1−42 oligomers. Reproduced with permission [125]. Copyright 2019, American Chemical Society. (**D**) (**a**) Raman spectrum of *Aβ*(1–42) in powder (black line) and SERS spectrum of this peptide at a concentration of 10^–6^ M (red line); (**b**) SERS spectra of different amyloids. Reproduced with permission [110]. Copyright 2021, American Chemical Society. (**E**) Average SERS (**a**) spectra of serum from healthy subjects (blue lines) and HD patients (red lines), including standard deviation (**b**). Reproduced from [126] with permission from the Royal Society of Chemistry.

Dopamine (DA). It is a well-known catecholamine neurotransmitter that is naturally synthesized in the brain and kidneys. It is found in the cerebrospinal fluid together with ascorbic acid, uric acid, serotonin, and epinephrine. The DA variation in the brain has been linked to several ND disorders. The band at 992 cm^−1^ was chosen as a marker band (Figure 4) and an LOD down to pM concentrations (as found in vivo in the cerebral extracellular fluid of patients) was detected [83].

Amyloid β. In AD pathology, *Aβ* aggregates, and its bioaccumulation is revealed by plaques that hinder the synaptic function and activate oxidative injuries at the neural level. SERS is both highly sensitive and selective at the molecular level; it can sense conformational changes in the secondary structure peptides adsorbed onto metallic surfaces [110]. However, there is still effort required for the comprehensive assignment of SERS profiles. The studies reported so far reveal that AuNPs can facilitate and control aggregation of *Aβ*, besides the fact that they are mostly used in SERS biomedical applications. Once again, the amide bands ascribed to the polypeptidic backbone chain are monitored (β sheets: amide III at 1230–1236 cm^−1^ and amide I at 1668–1677 cm^−1^; α-helix at amide III at 1268–1275 cm^−1^) [110].

Tau protein. In AD pathology, there is a significant increase in the phosphorylated tau versus total tau levels found in, for instance, saliva; therefore the ratio between these two values can be considered as a potential biomarker in saliva. On the contrary, in CSF this ratio does not show any increase in AD patients [123].

Prognosis for early stages or proneness to the onset of neurodegenerative processes can thus be assessed by using SERS-based approaches. The spectral window exploited for the detection of clinically relevant proteins and their monitoring is between 1000 and 1700 cm^−1^ (Figure 4).

The Raman/SERS-based approaches for detection of relevant biomarkers in ND diagnosis are summarized in Table 2.

**Table 2:** Specific neurodegenerative pathologies and Raman/SERS-based relevant strategies for their specific biomarkers’ detection and diagnosis [68].

ND	Protein Targeted for Diagnosis (Biomarker)	Raman/SERS Approach	SERS Platform Used for Detection
Prion disease	Misfolding of cellular prion protein (PrP^C^) into pathogenic form (PrP^Sc^) and PrP^C^ aggregation β-Sheets structure more present than α-helical structure in PrP variants	Raman—Monitoring the downshift of the amide II band from 1450 to 1555 cm^−1^ and the decrease in intensity for the amide III band during D_2_O solvent exposure related to concentration of S-fibrils [127] Analyzing the presence of the 1670 cm^−1^ band (amide I region) as an indicator of β-sheets structure, thus PrP^Sc^ formation, with 100% accuracy [78]	Direct deep UV resonance Raman analysis of pure S-fibrils and R-fibrils samples in mixtures with the D_2_O Direct Raman analysis of membranous blood pellet from scrapie-infected sheep (PrP^Sc^ was found to be located in blood fraction of lymphocytes)
	Changes in Cu (II) coordination inducing the formation of Cu (II)(His)4 complexes in weakly acidic pH → formation of PrP^Sc^ alters Cu(II) binding	SERS—Monitoring the free Cu(II) and Cu(II) bound histidine (Raman bands intensities I_1577_ vs I_1603_) [84] Monitoring the PrP^C^–Cu(II) coordination by quantifying the dependence between Cu(II)-binding and copper concentration [84,98]	SERS analysis of cell membranes: culturing the cells onto AuNP-film-based substrates (20–70 nm thick) self-assembled on glass from spherical NPs
Parkinson’s disease	α-Synuclein Decrease of dopamine levels during disease progression	Raman—Monitoring the heterogeneity of α-synuclein and conformational changes during fibrillation [85] SERS—Direct dopamine monitoring from CSF samples	300 µM concentration of protein required for thorough analysis Femtomolar levels of dopamine when using functionalized AgNPs
Huntington’s disease	Expression of the toxic mutant Hungtingtin (Htt) protein Peripheral fibroblast modifications caused by this pathology	Glutamine residues variability for monitoring (as polyglutamine) Raman—Spectral differences for fibroblasts that were known to be from a HD patient as compared with those coming from a healthy person	Spectral data collected originated from fibroblasts—extracted plasma membrane components associated to cholesterol and phospholipids as well as proteins containing tyrosine [128]
Alzheimer’ s disease	Amyloid-β(*Aβ*) and tau proteins Aggregation of the two proteins into extracellular amyloid plaques and intracellular neurofibrillary tangles Biochemical changes in the platelets collected from blood samples [106] Tears components analysis	Raman [79] and SERS [80]—Monitoring metal ions associated with amyloid protein aggregation: Zn(II) or Cu(II) bound histidine Raman bands Raman—Platelet alterations such as those related to amyloid precursor protein (APP) processing SERS—Indirect detection of tau protein by using magnetic hybrid NPs functionalized with specific antibody and Raman label (DNTB) [109] SERS—Physiologically relevant *Aβ* concentrations were investigated for determining the conversion from α-helical to β-sheet structure [117,129] SERS—Spectral response of tears collected from healthy subjects and known AD patients was compared in terms of protein component fingerprint; quantitative information related to pathological aggregates was assessed from the ratio I_1342_ vs I_1243_ [86]	Direct Raman analysis of metal coordinated complexes Increase in the intensity ratios for 758 cm^−1^ and 744 cm^−1^, assigned to the tryptophan sidechain and heme species specific to platelets Monitoring the SERS specific bands of DNTB at 1053 cm^−1^, 1332 cm^−1^, and 1553 cm^−1^ (fM levels) Microfluidic device enabling *Aβ* conformational biosensing as low as 10 fM [129], nanofluidic biosensor [130] Direct SERS analysis of *Aβ* by using chemoreceptor functionalized AuNPs-decorated polystyrene beads [108] Direct SERS analysis of tears components on a AuNPs-based substrate [86]

## 5. ZnO-Based Integrated SERS Biosensors—An Overview of Their Performance

Recently, new hybrid solid substrates based on ZnO and plasmonic materials were developed for SERS-based biomedical and clinical applications: for example, hybrid Au- or Ag-based ZnO substrates have been used for DNA detection at concentrations down to the μM level [111,131], whereas Ag/ZnO nanorod array/Ag substrates were applied to detect hemoglobin [132]. In another approach, Au/ZnO nanorods together with statistical analysis were used as a biosensing approach to diagnose interstitial cystitis/bladder pain syndrome in rats [133]. Furthermore, Kaminska et al. [134] fabricated a label-free SERS platform based on Au-coated ZnO films and used it to detect neopterin biomarkers from human plasma, an indicator for early diagnosis of malignant diseases with a detection limit of 1.4 nmol/L. Furthermore, an up-to-date review of ZnO-based substrates for SERS biosensing has been provided by Adesoye et al. [119]. Thus, we continue this section by surveying the most recent ZnO-based SERS biosensors with the capability for ultrasensitive detection of clinically relevant biomarkers and their monitoring for ND diagnosis.

First, we intend to offer an outline of the significant role of semiconductor (SC) nanomaterials, ZnO in particular, as enhancement substrates in various SERS applications. It is of utmost importance for the reader to firstly understand the optical phenomena involved in the SERS effect when solely SCs or SC-based hybrid nanomaterials are used as signal enhancers. Thus, we will start by presenting how ZnO/ZnO-based nanohybrids contribute to the Raman amplification signal of analytes.

**Figure 5:**
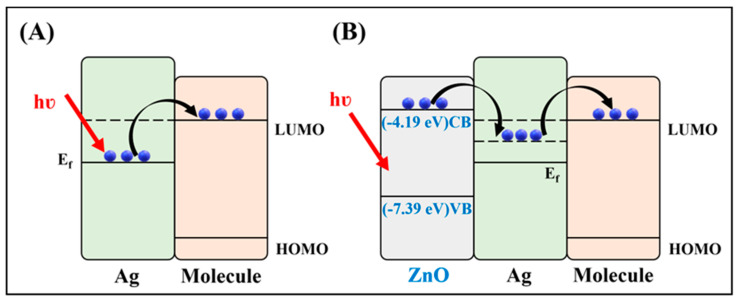
Schematic illustration of CT mechanism for Ag/molecule interaction (**A**) and ZnO/Ag/molecule interaction (**B**). VB: valence band, CB: conduction band, Ef: Fermi level, HOMO: highest occupied molecular orbital, LUMO: lowest unoccupied molecular orbital.

The enhancement mechanism for the SERS effect for ZnO alone or as a hybrid ZnO/metal substrate is certainly different and will be discussed from the two contributions specific to SERS: the EM and the CM. The EM is dependent on the “hot spots” provided by the substrate and was found to be higher for the hybrid ZnO/metal substrates due to the higher contact region between the metal and the ZnO nanostructures. The intimate contact of the analyte with the SERS-active substrate is therefore facilitated by expanding the contact surface area and boosting the “hot spots” density. From the CM point of view, there are demonstrated EFs for ZnO/Ag hybrid SERS substrates being at least one order of magnitude higher than for Ag-based SERS substrates [135]. This was justified as being a consequence of the contact between the semiconductor and the metal, driving towards an electron transfer from ZnO to Ag and an increased electron density for Ag. By fabricating SERS substrates composed of noble metals and amorphous semiconductors, there are benefits coming from sensitivity, selectivity, and stability viewpoints. A schematic diagram shows the charge transfer (CT) contribution and subsequent chemical enhancement (Figure 5). However, there is evidence showing that this effect is normally limited to about one order of magnitude for metallic nanostructure-based SERS detection of bioanalytes [10]. Surprisingly, in the case of semiconductor–molecular interactions, there are options for optimizing the CT effect, such as in the case of graphene, by facilitating interfacial processes such as surface electron transfer that is correlated to the metastable electronic state in ZnO [119]. In this context, a remarkable SERS EF of 10^5^ grace to a significant CT effect was demonstrated for amorphous ZnO in the shape of nanocages [136]. Thus, for ZnO-based SERS substrates one can manipulate the SERS activity by shifting to a predominant CM attributed to the high efficiency CT between the semiconductor substrate and the analyte.

The advantages of this novel class of enhancing nanomaterials are well known: they offer spectral reproducibility and are chemically stable, biocompatible [137], controllable during fabrication, affordable, reusable [132,138], and recyclable [139]. Subsequently, we will summarize, without going into too much detail (as there are several well-documented reviews already available (see Table in Section 6)), the design and fabrication of ZnO nanomaterials in combination with plasmonic materials that have been tested and have already demonstrated their high SERS performances.

ZnO-based materials exhibit excellent physical properties, such as a wide bandgap (3.2 eV) and flexibility in fabrication, are affordable, and have a high surface-area-to-volume ratio. Thus, from the manufacturing viewpoint, exploiting the huge potential of such special materials was reported in several applications. For instance, by tuning the morphology, alignment, and the density of the ZnO-based nanorods (NRs), the SERS signals were enhanced with an EF of ~10^5^ [140]. In this case, the vertically aligned ZnO nanorods were covered with AgNPs in various amounts and finally, the EF obtained was practically comparable with the one reported by Wang et al. from ZnO-only nanocages [136]. Parameters such as synthesis time, deposition time, and their corresponding density of nanostructures per surface area generate the highest SERS enhancement observed in each study, providing practical strategies for an increased density of plasmonic hot spots and an extended area for the deposition of analyte molecules covered by such regions of strong near-field intensity. The intrinsic property of ZnO nanocrystals for boosting the SERS detection efficiency solely via chemical enhancement was demonstrated unequivocally by Wang et al. [141].

From here on we will focus mainly on the salient features of newly designed ZnO-based nanomaterials that have shown practical use in SERS diagnosis. To our knowledge, this would be the first review that highlights the practical use of ZnO-based SERS biosensors in clinical applications.

To efficiently enhance the Raman signal, ZnO-based materials are mostly used together with Ag or Au. The SC–metallic synergistic design attains improved properties, such as high electromagnetic (from the metal) and charge transfer (ZnO) contributions to the overall enhancement, plus a complementary absorbance due to the local surface plasmon resonance that takes place at the junction spots between these materials. Moreover, an additional reason for the coupling with a noble metals lies in the localization of the surface plasmons in the visible and near-infrared regions that are valuable in vibrational-based biosensing [142]. The potential of SCs for generating enhanced Raman signals was demonstrated in 1982, when Yamada et al. [143,144] detected the SERS spectrum of pyridine on single crystals of NiO and TiO_2_. This was the starting point of when SC materials and SC-based hybrid materials became popular in SERS applications. Semiconductors, such as ZnO, in combination with plasmonic materials have an important contribution to gigantic SERS effects due to some of their properties: excellent physical and optical properties (strong light confinement and high refractive index value, which enhances the SERS signal [145]), a wide bandgap (3.37 eV), and a high surface-area-to-volume ratio [119].

Moreover, ZnO is considered cheaper and easier to produce in high amounts; it is also far more stable when exposed to air in comparison with Ag and Au and reusable thanks to its photocatalytic activity [146]. When ZnO is used as a SERS nanostructured substrate, the mechanism of light enhancement is complex and is directly dependent on its SC and piezoelectric dual properties. Optical effects such as light absorption and trapping, photo-induced charge transfer, and optical resonance can occur [147]. Generally speaking, the enhanced Raman signal between the analyte and the ZnO substrate has been mainly assessed with a large surface-to-volume ratio and a charge transfer between the two components [142]. However, one drawback lies in the frequencies at which the plasmonic resonance occurs being far from the used excitation sources [148]. Another limitation is related to the high solubility in acidic conditions. This is of crucial importance in the case of ZnO/Au hybrid nanomaterials in which tetrachloroauric acid (HAuCl_4_) is used as a precursor for Au nanoparticle (NP) synthesis [149]. Excellent reviews detailing the SERS enhancement mechanisms in three classes of SC materials (inorganic SCs, metal/SC composites, and organic SCs) exist in the literature [150,151]. Another review by Krajczewski et al. [152] focuses on the preparation and characterization of SERS nanostructured non-metallic materials, including ZnO nanowires, nanorods, nanotrees, and nanoneedles covered by Ag or Au. Their SERS enhancement capabilities were tested by using well-known testing analytes such as R6G, Sudan II and IV dyes, and methylene blue, with LODs down to 10^−12^ M. In their review, Marica et al. [153] covered the main recent results of ZnO-based nanostructures used for fluorescence and Raman signal enhancement. The improved SERS capabilities of this class of nanomaterials are highlighted based on the detection of R6G using various designed ZnO-based SERS hybrid substrates, with LODs up to 10^−13^ M and EFs of 10^8^.

ZnO-hybrid SERS substrate fabrication has advanced to the development level of obtaining 3D multilayered SERS substrates that provide additional plasmonic hot spots in the z-axis, a key asset in biosensing. The components of a 3D substrate are highly tunable from the sub-micrometer to centimeter scale and can be obtained by the joint use of various top-down and bottom-up fabrication techniques, such as nanolithography, evaporation deposition, self-assembled-based methods, chemical and catalytic processes, etc. Although some of the fabrication techniques are not cheap, they provide the possibility for new 3D SERS detection platforms adaptable to different sensing settings, including dynamic detection in microfluidic channels. A robust optimization of every fabrication step will provide scalable SERS substrates with undeniable advantages over two-dimensional ones. An excellent critical review of pure ZnO/hybrid nanostructures with SERS applications has been recently published [119]. It covers achievements in the SERS detection of several probe molecules with well-known spectral fingerprints: methyl orange/blue, methylene blue, rhodamine B/6G (R6G), bradykinin, p-aminothiophenol, thiophenol, and erythrosine B, with LODs down to 10^−14^ M and EFs up to 10^11^.

Starting in 2011, the interest in hybrid nanomaterials based on ZnO films or hierarchical structures has increased and demonstrated their great potential in the SERS detection of contaminants and adulterants at trace levels.

**Figure 6:**
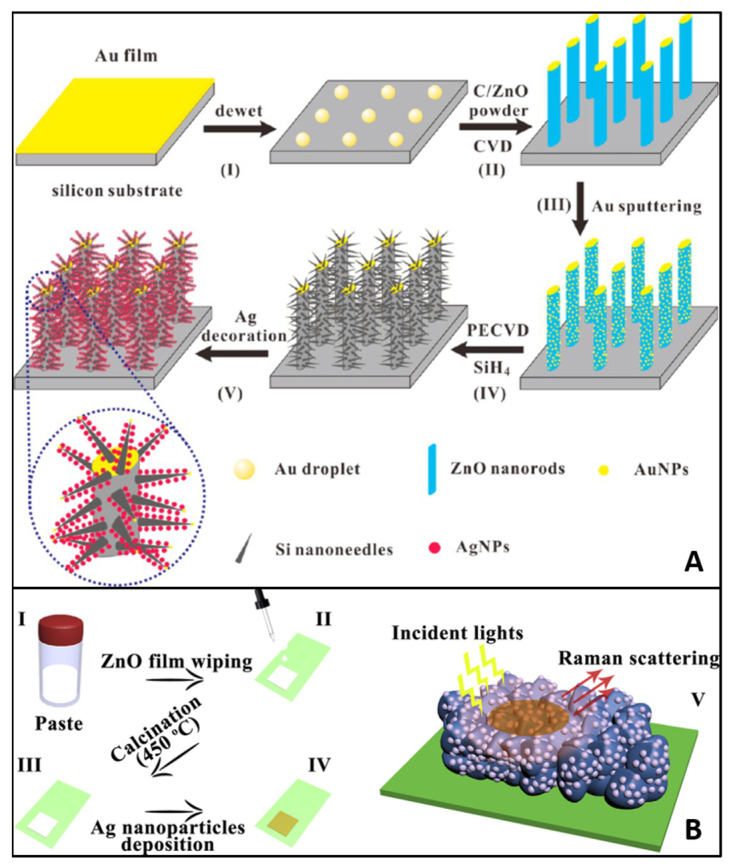
(**A**) Schematic protocol showing the intermediate steps for the fabrication of 3D ZnO/Si nanomace arrays decorated with Ag NPs. Reproduced with permission [154]. Copyright 2015, American Chemical Society. (**B**) The design and fabrication of the 3D porous Zn/Ag SERS active substrate (I–IV: preparation process of the substrate; V: final structure of the substrate). Reproduced with permission [155]. Copyright 2017, Elsevier B.V.

In 2015, Huang et al. [154] reported, for the first time, the fabrication of a 3D substrate based on ZnO and Ag with direct real-life application: melamine detection in pure milk. The first step was the growth of ZnO nanorods on silicon (Si) wafers by using a vapor−liquid−solid deposition technique and sputtering of an Au film. Secondly, the Si nanoneedles were grafted by means of chemical vapor deposition resulting in hybrid ZnO/Si nanomace arrays. Finally, the Ag NPs were immobilized onto the 3D structures by a galvanic displacement reaction. The fabricated 3D hierarchical nanostructures and the fabrication process are graphically presented in Figure 6A.

In 2017, Wang et al. [136] reported, for the first time, the SERS activity from of a SERS amorphous SC nanomaterial in the form of ZnO nanocages that had an EF up to 10^5^, which was higher than the crystalline counterpart. This newly designed nanomaterial was chemical synthesized, followed by dehydration and calcination steps. The SERS sensitivity and stability were tested towards several mercaptoanalytes. The authors assigned the remarkable SERS abilities to the interfacial charge transfer process between the ZnO nanocages and the analyte that resulted from the metastable electronic states of the amorphous ZnO nanocages. Additionally, a joint use with a plasmonic material such as Ag or Au can produce higher EFs than those produced exclusively by Ag or Au, with lower costs.

It is worth mentioning the 3D porous ZnO/Ag SERS designed nanomaterials reported by Yang et al. [155], which also exhibited both ultrasensitivity in detecting R6G (LOD of 10^−11^ M), CV, malachite green, and Sudan I and the possibility of entirely degrading the target molecules in situ under UV light for a fully reusable SERS substrate (Figure 6B).

Before concluding with challenges and limitations specific to Raman/SERS-based approaches for the detection of relevant biomarkers in ND diagnosis, we summarized in Table 3 the most relevant strategies for clinical practice based on ZnO hybrid substrates.

## 6. ZnO-Based Biosensors for the Ultrasensitive Detection of ND Biomarkers

**Figure 7:**
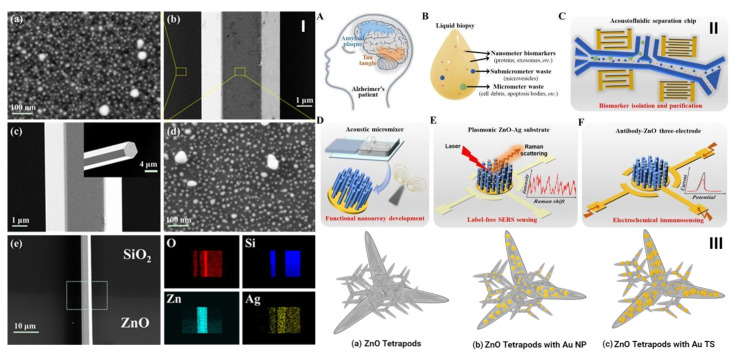
(**I**). (**a**,**d**) Enlarged regions of the ZnO microrod and film from (**b**), the SEM image of 20 s sputtered time hybrid Ag/ZnO substrate with the ZnO/Ag hybrid microcavity. Representative SEM images showing: (**c**) a single ZnO microrod and (**e**) the individual ZnO microrod on the SiO_2_ substrate partially sputtered with ZnO film, with (bottom right) the EDS mapping of the elements Si, O, Zn, and Ag on the microrod and substrate. Reproduced with permission [19]. Copyright 2016, AIP Publishing. (**II**). An acoustofluidics multimodal diagnostic platform to isolate and detect the AD markers *Aβ* and *tau*. The images (**A**,**B**) illustrate the liquid biopsy protocol employed to analyze the circulating biomarkers, whereas (**C**–**F**) reveal the design of the separation chip, the nanoarray development, the label-free SERS sensing phenomenon, and the electrochemical immunosensing. Reproduced with permission [13]. Copyright 2022, Elsevier B.V. (**III**) Light-assisted prepared SERS-active ZnO TPs decorated with branched AuNPs. Schematic view of as-grown (**a**) ZnO TPs; (**b**) ZnO TPs after photochemical functionalization with Au spherical NPs; (**c**) ZnO TPs after seed-mediated growth of branched NPs (inspired by the SEM micrographs included in [149]).

Another application area in which ZnO-based hybrid materials are currently popular is related to neurological disorders (Table 3). Neurotransmitters such as DA, serotonin, acetylcholine, GABA, and glutamate are mainly associated with NDs such as AD, PD, schizophrenia, social anxiety, attention deficit hyperactivity disorder, HD, bipolar disorder, and restless leg syndrome [20,116]. The assessment of the level of these indicators in biological systems can offer valuable insights for the diagnosis and management of NDs. An early study conducted by Lu et al. [19] reported the detection of DA at concentrations down to 10^−12^ M by using a hybrid ZnO/Ag microcavity based on the whispering gallery mode effect (Figure 7I). In such a microcavity, the light propagates in circulating waveguide modes due to multiple internal reflections, which translates into high enhancement factors and minimal signal loss [19]. The microcavity existed in one selected ZnO microrod with hexagonal cross section and a diagonal of 6 μm that was synthesized by a simple vapor-phase transport process and placed on a SiO_2_ substrate. The AgNPs were deposited on the microrod by the sputtering ionic beam sputtering method.

The very recent work of Hao et al. [13] proposed an integrated acoustofluidic system based on ZnO nanorods and Ag NPs with triple function for the ultrasensitive and rapid detection of AD biomarkers from human plasma. The proposed platform is shown in Figure 7II and has demonstrated promising results for the early diagnosis of AD. The fabricated multidevice can isolate and purify AD biomarkers such as *Aβ* peptides and tau proteins for an improved signal-to-noise ratio. The integrated acoustofluidic mixing-based multimodal biosensor (SERS and electrochemical immunosensor) was fabricated by in situ nanopatterning with a ZnO nanorod array decorated with Ag NPs.

Another 3D ZnO-based hybrid nanomaterial employed for the SERS detection of apomorphine, a drug used for the management of PD symptoms, with an LOD of 1 μM (0.27 μg/mL) was reported by Picciolini et al. [149]. The group fabricated 3D SERS substrates made of ZnO tetrapods decorated with branched Au NPs using a photochemical multistep approach (Figure 7III). The ZnO tetrapods were fabricated by applying a multigram scale synthesis, whereas the Au seeds were chemically grown onto the ZnO structures by photoreduction. The SERS substrate was centrifuged on a Si wafer, and the growth of the Au NPs was strictly controlled.

Hybrid ZnO-based SERS biosensors with clinical potential exploit a range of laser lines as excitation sources; the essential technical parameters are mentioned in each case and is a useful guideline for experts that are researching the subject. We summarized all these details below for comparison and a clear overview in Table 4 and Table 5.

## 7. Conclusions and Future Perspectives

As shown in the last subsection, the incorporation of ZnO-based solid substrates in SERS detection platforms for the SERS diagnosis of NDs is still at its beginnings. However, this is a promising avenue of research considering the high impact and performance of the current biosensing approaches. A literature survey based on “SERS” and “ZnO” keywords yielded 100 studies (including reviews) from the last decade, from which nearly 60% have focused on materials science research, such as novel hybrid ZnO substrate fabrication, optical characterization, and SERS performance. Another 30% exploited these plasmonic materials’ potential in diagnosis applications, whereas the rest reported on doping effects, photocatalytic properties, and the charge-transfer resonance effect. The clinically relevant approaches report reliable figures of merit for detecting glucose, melamine as an adulterant, cancer- and inflammation-related biomarkers, human aqueous humors, exosomes, DNA, oligonucleotides, and bacteria. These studies reflect the wide range of biosensing purposes already found as suitable for ZnO-based SERS substrates and their huge potential for future biomedical devices designed for early diagnosis.

However, there is a long road ahead to address all the challenges derived from SERS methodology. The fluctuations in the SERS signal can be limited by strict control over the fabrication process of the substrates and their stability. By only improving the detection sensitivity and limiting its dependency on the intrinsic enhancement of the substrates, the reproducibility and the interference reduction will speed up its clinical adoption.

**Figure 8:**
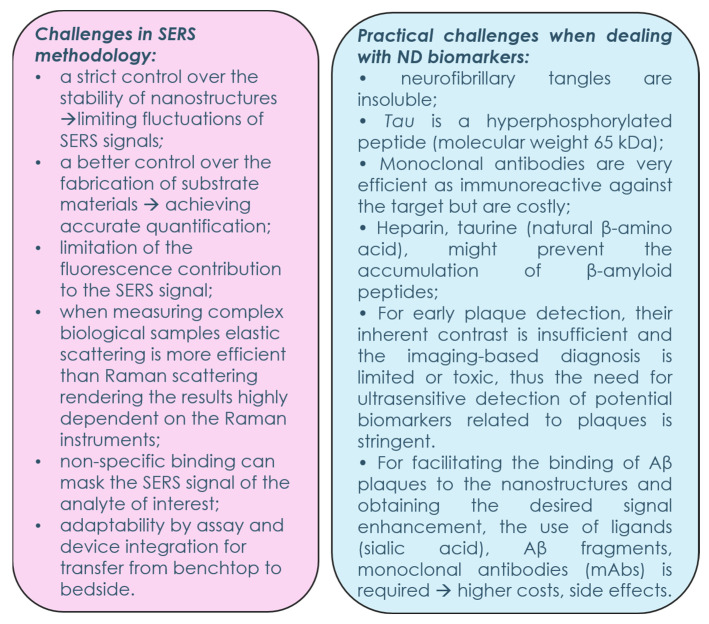
A diagram summarizing the main practical challenges when using SERS biosensors for the detection of ND biomarkers.

Standardization of spectra acquisition for universal applicability, independent of on-site or within-lab sampling of biomarkers and for quantitative determination from the spectral output, is also a prerequisite for translating the methodology into clinical premises. Additionally, data analysis plays an important role in obtaining a fast spectral pathology due to the huge number of spectra obtained that require unbiased handling and selective analysis in the relevant spectral region (i-PCA [86]) or by using automated trained models for distinguishing between healthy and diseased cells/tissues. For up-to-date supervised models (machine learning, deep learning), big datasets are necessary for training and validating the analysis models. Moreover, by using multivariate analysis the cost issues generated for employing antibodies might be circumvented and the simpler, reagent-minimal SERS-based platforms could become more reliable in practice. Thus, several challenges are needed to be overcome for the future progress of SERS biosensors towards clinical practice, as shown in Figure 8.

### Perspectives

A promising trend is represented by ZnO-based hybrid SERS substrate fabrication, which has advanced to developmental level. Moreover, three-dimensional, multilayered SERS substrates provide additional plasmonic hot spots in the z-axis, these being of key importance in boosting practical performance. Usually, this superior yield is obtained by creating periodical nanopatterned arrays on solid supporting substrates. Although some of the fabrication techniques have high costs, they also provide the possibility for new 3D SERS detection platforms to adapt to different sensing settings, even dynamic detection in microfluidic channels. Moreover, a robust optimization of every fabrication step will provide scalable SERS substrates with undeniable advantages over two-dimensional ones. In-depth irradiation will provide a larger hot spot volume, whereas nanopatterning will increase the active detection area, which will translate into more analyte–plasmonic particle interactions and improved detection sensitivity. The laser wavelength or instrument specific parameters will have a lower impact on the overall SERS enhancement due to the higher density of hot spots along the z-axis.

Recently, sialic acid has been considered a suitable candidate for nanoplatforms designed with a functionalized layer that is meant to bind the *Aβ* fibrils due to its biocompatibility and high affinity. In vivo studies have already assessed sialic acid as the capturing element for elaborated detection platforms designed for AD diagnosis or as engineered drug delivery systems [105].

In conclusion, the design and fabrication of 3D ZnO-based platforms will pave the way for the SERS technique to become a multipurpose method with an important role in the future development of medical biosensors.

## Figures and Tables

**Table 1:** Techniques used for ND diagnosis with their known assets and limitations from practical point of view [67,68].

Techniques	Clinical Examples	Advantages	Disadvantages	Current Use
**Imaging techniques**	**Magnetic resonance imaging (MRI)**	Label based Reliable High resolution Wide collection of fluorophores or contrast agents	Incapable of differential diagnosis due to chemical contrast limitations Current need for tissue-specific labeling Toxicity due to dye usage	*In vivo*
	**Optical coherence tomography (OCT)**			*In vivo*
	**Two-photon excited fluorescence (TPEF)**			*In vivo* (limited)
	**Photoacoustic imaging**	High sensitivity Specificity Rapid output	Costs due to specific labels Specific absorption limitations of dyes in practice Toxicity due to dye usage	*In vivo*
	**Confocal fluorescence microscopy**			
**Spectroscopic techniques**	**IR microscopy**	**Label free** Provides chemical contrast due to molecular vibrations specific selectivity	Long wavelength → low spatial resolution Interference of water absorption specific to biological samples Tissue depth penetration is limited	*Ex vivo*
	**Raman scattering**	**Label free** Complex matrices as samples with minimal preparation required (biofluids, cells, tissues, etc.) No interference from water (intrinsic to samples) Selective tool for NDs differential diagnostics Imaging of (bio-)molecular distribution Specific molecular fingerprinting spectral output Clinical adapted portable setups: optical fibers with possibility to integrate with cannulas, endoscopes, catheters	Time consuming when using mapping technique of large tissular areas Low efficiency of the scattering process translated into inherently weak Raman signals Potentially destructive to the sample due to long laser exposure times	*In vivo*
	**Hyperspectral Raman imaging**	Able to show the amyloid plaques, neuritic plaques, or neurofibrillary tangles and is able to distinguish tissue components for margin determinations		
	**Surface-enhanced Raman scattering**	**Label free** No need for staining High chemical specificity Combined with cryo-sampling of brain tissue	Signal enhancement is local, mainly due to the molecules in close contact with the metallic nanostructures	*In vivo*
		**Label based** When combined with *spatially offset (SORS)* the detection is possible within the skull Bio-barcode assays for differential PD diagnosis Reduced time Multiplexing capacity	Silver nanoparticles (AgNPs) are shown to alter neurotransmitters in in vivo conditions (rats) Reliant on costly antibodies specific to NDs	
**Molecular Biology techniques**	**ELISA/Western blot**	**Label based** High accuracy Suitable for routine protocols (predictive and diagnosis screening)	Costly due to highly specific reagents required, invasive, and laboratory dependent Time consuming when including bioinformatics protocols	*Ex vivo*
	**Immunohistochemistry**			
	**Genomics** **(PCR, RT-PCR, DNA sequencing, (epi)transcriptomics)**			

**Table 3:** ZnO hybrid substrates and their practical performance in clinical practice.

Analyte Neurotransmitter Biomarker	SERS Substrate	Practical Advantages	LOD/EF	Reference
**Crystal violet** **rhodamine R6G**	ZnO-based superstructure highly ordered (flower-like 3D ZnO arrays on glass substrate decorated with self-assembled Ag NPs) via induced photochemical reduction.	Multicomponent detection ability, needed for monitoring multiple metabolites in biofluids.	**10^−10^ M**	[156]
**Crystal violet** **rhodamine R6G**	Ag/AgBr/ZnO film prepared on a conventional glass substrate to regenerate by a visible-light-driven photocatalytic process.	High yield of reusability (up to eight times without losing the SERS efficiency).	**5.95 × 10^−13^ M /10^9^ 1.46 × 10^−11^ M/10^8^**	[138]
**Melamine**	Nanonecklaces of ZnO in combination with 45 nm Au thin films, deposited using a chemical vapor deposition technique (grown faceted and one directional ZnO nanonecklace arrays on r-plane sapphires).	Ready-to-use substrate for integrated biosensing applications. Strict control over the density and “hot spot” distribution. The faceted substrate yields higher electromagnetic enhancement and higher free energies.	**10^−5^ M/10^4^**	[157]
**Methylene blue**	ZnO nanorods coated with Au by means of a low-temperature hydrothermal route and sputtering deposition of Au nanoislands.	By taking advantage of the photocatalytic properties and inducing the degradation of analytes when exposed to UV, this approach provides substrates that are recyclable and affordable and have high reproducibility of SERS spectra and a long shelf life.	**10^−12^ M**	[139]
**Melamine adenine**	Superhydrophobic substrate that consists of arrays of ZnO nanorods coated with Ag NPs.	The superhydrophobic condensation effect had a massive contribution to the SERS signal (high dependency of the Raman amplification signal on the water contact angle and the water droplet volume, which are controllable).	**10^−6^ M/not given** **10^−6^ M/not given**	[111]
**Crystal violet** **melamine detection in milk**	3D substrate based on ZnO and Ag for direct SERS detection in real-life applications (Ag NPs/ZnO nanorods/Si nanomace arrays).	Increase in hot spot density by an additional expansion of the hot spot arrangement along the third dimension. Practical applications in food and environmental safety.	**10^−16^ M** **10^−10^ M/** **10^7^**	[154]
**Benzodiazepines (BZDs) in mice;** **serum** **Estazolam in urine and serum samples**	Cabbage-like (111) faceted Ag nanosheets decorated with ZnO NPs	Successful group-targeting screening of 5 BZDs (estazolam, oxazepam, alprazolam, triazolam, and lorazepam) and their concentration changes during metabolic process in mice serum.	**0.5 nM**	[121]
**Dopamine**	Hybrid Ag NPs-decorated ZnO WGM microcavity/SiO_2_ Relevant SERS marker bands: 675, 780, 1150, 1280, 1350, 1450, 1497, 1580 cm^−1^.	Strong confinement and light enhancement inside the microcavity due to the effect of an optical resonant cavity.	**10^−12^ M**	[19]
**Dopamine**	Matchstick-shaped Au-ZnO nanorods Relevant SERS marker bands: 1363, 1583, 1765, 1904 cm^−1^.	Facile one-pot colloid synthesis method. High reproducibility after visible-light-assisted cleaning. Promising biocompatibility and recyclable SERS detection platforms for various molecular species.	**10^−5^ M/1.2 × 10^4^**	[158]
**Bombesin**	Electrochemical ZnO NPs ZnO NPs from banana skin Relevant SERS marker bands: 638, 760, 1011, 1338, 1438, 1546 cm^−1^ and 1062, 1129, 1295, 1438, 1462 cm^−1^.	Biomarkers naturally found in body fluids and indicators of malignant tumors such as glioblastoma or pancreatic, stomach, or breast cancers.	**3 × 10^−5^ mol/L/10^3^**	[118,120]
**Neurotensin** **(neuropeptide modulator), bradykinin**				
***Aβ* samples**	Multimodal (SERS and electrochemical) biosensor with integrated acustofluidics-based on ZnO nanorod @Ag NPs patterned on a Au electrode. Relevant SERS marker bands: 442,491, 569, 677, 718, 776, 793, 842, 954, 1001, 1069, 1139, 1163, 1187, 1216, 1252, 1311, 1355 cm^−1^ and their intensity fluctuations.	Ready-to-use, integrated clinically, accurate, sensitive, and rapid biosensor for AD biomarkers from human plasma.	**120 fM/7.5 × 10^5^**	[13]
***Aβ* from human plasma (10 AD patients and 7 healthy controls)**			--/--	

**Table 4:** Comparison of SERS methods for ND biomarker detection using the ZnO hybrid SERS substrates.

Detection Substrate	Detection Setup	Detected Biomarker	LOD	Reference
ZnO/Ag hybrid WGM microcavity	Lab RAM HR 800 micro-Raman system with an excitation of **514.5 nm**	Dopamine	1 pM	[19]
ZnO-Ag nanoarray SERS substrate	HORIBA Jobin Yvon Raman spectrophotometer equipped with an Olympus BX41 microscope; excitation: **785 nm**	AD biomarkers containing *Aβ*_42_	120 fM	[13]
ZnO tetrapods decorated with branched AuNPs	Aramis Raman microscope from Horiba Jobin-Yvon equipped with a He-Ne laser: **633 nm**	Apomorphine, a dopamine agonist and cancer cells	1 μM (0.27 μg mL^−1^)	[149]
Matchstick-shaped Au–ZnO heterogeneous nanorods	Renishaw Raman system inViaReflex spectrometer LabRam HR 800 spectrometer of HORIBA. (**532 nm**, **632.8 nm**, and **785 nm**)	Dopamine	10^−5^ M	[158]
3D hierarchical substrates with ZnO nanowires (NWs) on ordered vertically aligned Si NRs decorated with AgNPs	Jobin Yvon high-resolution Evolution 2 system, excitation laser: **532 nm**	Conformational change of human islet amyloid polypeptides (hIAPP) specific to NDs or type II diabetes	10^−8^ M (single amyloid aggregate level)	[159]

**Table 5:** Key aspects and novelty of this review as compared with previously published reviews on the topic of ZnO hybrid substrates for SERS-based ND diagnosis.

Recent Reviews on a Similar Topic	Spectroscopies Involved	Performance and Clinical Use	Practical Key Aspects	Limitations	Originality
Raman Spectroscopy: An Emerging Tool in Neurodegenerative Disease Research and Diagnosis; Devitt et al. [68]	Resonance Raman scattering Stimulated Raman scattering CARS, SERS Raman-based microfluidic devices	Through comprehensive subsections, clinically relevant spectral features are indicated for each prevalent ND along with their interpretation.	Selected pathologies are described with spectral read-out options by using technologies that are widely available. SERS label-based detection strategies only are debated.	Indirect detection involves particular species that could interfere with the targeted spectral features or exhibit toxicity.	Good assets and drawbacks in practice with wide implementation. For Raman-based testing: amyloids are the prominent biomarker surveyed.
ZnO Nanostructured Based Devices for Chemical and Optical Sensing Applications; Sha et al. [160]	Field effect transistors (FET) for enzymatic sensing Electrochemical sensing Photoelectrochemical sensing	Valuable clinical information is summarized by targeting biorecognition of neurotransmitters, proteins, and biomarkers for other diseases and their sensing performance. Chemical sensing applications with wide biomedical impact are detailed.	Progress towards biomedical and optical sensing: ZnO sensors are examined for their merits. Fundamental ZnO-sensing mechanisms are explained for a better insight of how to exploit their special properties. Environmental applications are also included: solar cells, photodetectors, etc.	Practical issues are described in detail, along with the specific solutions for further development. Main applications rely on the ZnO hybrid NPs type of nanomaterials Other downsides: lab-research level, only partially integrated into clinical devices, not affordable.	Focus on the devices containing 2D ZnO-based nanomaterials. No Raman- or SERS-based sensors are included.
ZnO and TiO_2_ Nanostructures for Surface-enhanced Raman Scattering-Based Bio-sensing: A Review; Adesoye et al. [119]	Biosensing and biodetection by using ZnO- and TiO2-based hybrid SERS substrates	Strategies for cell imaging, screening of DNA, proteins, and pathogens are offered. From small target biomolecules to macromolecules and cells, the whole area of biosensing for chronic disease diagnosis is covered.	Main fabrication techniques are listed and pure ZnO/ TiO2 or hybrid nanomaterials are recommended for biosensing. ZnO- and TiO2-based substrates are discussed for the SERS detection of DNA, clinically relevant proteins or lipids, and cell imaging.	SERS capability of electrochemically synthesized pure ZnO NPs monitor the treatment of neurotransmitter-dependent diseases. No technical details for NDs diagnosis or specific biomarkers detection and monitoring.	Wide variety of synthesis methods, design with special coatings, and integration of ZnO- and TiO2-based SERS biosensors. Biological applications with clinical relevance are detailed.
Salivary Biomarkers for the Diagnosis and Monitoring of Neurological Diseases; Farah et al. [123]	Genomics Proteomics Metabolomics	The salivary biomarkers for early diagnosis of NDs are discussed as reliable and non-invasively tested indicators for normal/abnormal physiological and neurotoxic functions.	Easily accessible biofluid Omics-based technologies promoted as PoC routine analysis solutions. Alone, salivary biomarkers will not be able to diagnose NDs but could serve as a helpful initial test.	Main challenges in practice are the costs. Some of the ND biomarkers did not show modified levels in saliva, only in other biofluids.	Their unilateral discussion is based on the omics clinical diagnostics of NDs. No spectroscopic-based options are included.
On-Chip Detection of the Biomarkers for Neurodegenerative Diseases: Technologies and Prospects; Song et al. [161]	Chip-based technologies for ND biomarker detection: fluorescence, micro-cantilever deflection, chemiluminescence, surface plasmon resonance, reflectometric interference, and other optical sensing techniques	The ultrasensitive screening of biomarkers in serum, CSF, tears, or other biofluids is the way towards clinical validation. Due to the size of the protein biomarkers, nanostructure-based biosensors are most suitable for achieving the ultrasensitivity suitable for PoC settings directly from biofluids.	Bioaccumulation and aggregation mechanisms are thoroughly discussed. Inconsistency among different biofluid tests are highlighted for chromic, progressive ND approaches showing pre-clinical results.	Summarizes recent immunoassays-based sensing towards clinical integration by mainly relying on the NP-type nanomaterials. ZnO-based integrated substrates are not shown.	Pros and cons for PoC NDs diagnostic for bedside and source-limited settings: electrochemical, fluorescence, chemiluminescence, or plasmonic response, integration into miniaturized devices. Few Raman/SERS-based approaches are mentioned.
Raman Spectroscopy Techniques for the Investigation and Diagnosis of Alzheimer’s Disease; Polykretis et al. [162]	SERS, CARS Tip- or fiber- enhanced Raman spectroscopies (TERS or FERS), Non-linear techniques such as coherent Raman scattering	Key species associated with ND, their structure and aggregation process, and secondary structures of different amyloid-β peptides were inspected by SERS to correlate the structural rearrangement processes involved in self-aggregation and fibrillation.	Practical key aspects of the Raman-based techniques for the investigation and diagnosis of AD are detailed: minute amounts of sample required, label-free detection, versatility, sensitivity, and cost convenience.	Physical principles, benefits and drawbacks, target samples and concentration/dimensional range are tabled for Raman-based AD diagnosis. ZnO-based integrated substrates are not shown.	The scope of the review is to discuss recent advancements in Raman-based investigation and diagnosis of AD, highlighting their potential in monitoring the fingerprint of AD-specific biomarkers from biological samples (even brain tissue) and distinguishing between healthy and AD patients; very promising in clinical setting
Raman Spectroscopy and Neuroscience: from FundamentalUnderstanding to Disease Diagnostics and Imaging; Payne et al. [163]	Spatially offset Raman scattering (SORS and SESORS) CARS, SERS, TERS Stimulated Raman scattering Resonance Raman scattering and SERRS	Oxidative stress monitoring and CARS imaging are discussed. Raman-based probes are already implemented in surgery to remove brain tumors and collect data in real-time for PoC device integration.	Multiplexing capability of SERS for ND diagnosis device development is introduced. Label-based technologies are mainly discussed, proving high performance in clinical diagnosis.	Raman-based techniques are discussed combined with other conventional methods (MALDI-TOF MS, FTIR, or electrochemical based) ZnO-based integrated substrates are not shown.	Diseases discussed include neurotrauma, neuroinflammation, and neurological cancer alongside neurodegeneration. Mean SERS spectra of the four regions of the brain are exhibited and interpreted.
Emerging Two-Dimensional Materials-Based Diagnosis of Neurodegenerative Diseases: Status and Challenges; Wu, Dong et al. [164]	Field effect transistors (FET), SERS, electrochemical, photoelectrochemical, fluorescence, molecular biology conventional methods	Combining the nanomaterial-based signal amplification and electrochemical sensing provides improved sensitivity and selectivity. Challenges in obtaining reproducibility, for instance for the ratiometric electrochemical DNA nanosensor.	Challenges identified by the authors are related to material-specific performance, false positive or negative issues, stability of graphene-based materials in vivo due to biomolecular interactions and hydrophobicity of graphene, and, last but not least, the costs of such biosensors.	Recommended are mainly graphene or graphene-oxide-based 2D materials for SERS quantitative analysis of ND biomarkers. Other sensing methods: photo/electrochemical, immunoassays, fluorogenic, etc. Only ZnO-graphene integrated substrates are shown.	Promising SERS strategies with capability to integrate 2D materials and biomolecules for ND diagnosis and ultrasensitive detection of biomarkers.
This work	Raman- and SERS-based ND diagnosis and monitoring of specific biomarkers. Rational evaluation of the up-to-date ZnO-based hybrid SERS approaches for biosensing.	Focus on the potential of high-performance SERS analysis to reflect the early stages of NDs or the progression of disease. Conventional methods for early diagnosis of NDs are detailed along with their limitations.	Essential SERS spectral features extraction, relevant in ND monitoring and clinical decisions. ZnO-based hybrid substrates with promising sensitivity and some with relevant clinical results are discussed.	Practical key aspects for designing high-performance ZnO hybrid SERS integrated biosensors for the detection of ND biomarkers.Biosensing challenges in practice and possible solutions.	Comprehensive insight on SERS biosensing ND biomarkers as a clinical tool from the current TRL. SERS-based profiling of proteinopathies.

## Data Availability

The data presented in this review are available on request from the corresponding authors.
